# The 7 Muses of Neuro-Creative Cycle: How some patients with Parkinson's disease can unleash latent creativity

**DOI:** 10.3934/Neuroscience.2025014

**Published:** 2025-06-23

**Authors:** Kyung Hee Kim

**Affiliations:** Department of School Psychology and Counselor Education, William & Mary, 301 Monticello Avenue, Williamsburg, VA 23187, USA

**Keywords:** 7 Muses of the Neuro-Creative Cycle, Parkinson's disease, artists, dopamine therapy, independence, curiosity, playfulness, confidence, openness, interdependence, passion

## Abstract

Although dopamine therapy is known to enhance creativity in some artists with Parkinson's disease (PD), similar creative changes have been observed in individuals with other neurological conditions, even without such treatment. This suggests that broader neurological and psychological factors beyond dopamine alone may influence creativity in PD. In this paper, I examined these influences through the lens of the *7 Muses of the Neuro-Creative Cycle*: independence, curiosity, playfulness, confidence, openness, interdependence, and passion. Originally developed to support creativity in healthy individuals, the 7 Muses framework aligns closely with the structural and functional brain changes, as well as psychological shifts, commonly seen in PD. These changes may unlock latent creative potential, enabling PD artists to express themselves more authentically. By promoting a dynamic balance between top-down (goal-directed) and bottom-up (emotion-driven) processing across the creative cycle, preparation, imagination, and verification, PD-related changes may help reduce self-censorship, foster originality, and support the creation of meaningful, valuable work. Ultimately, beyond the effects of dopamine, some PD patients may awaken their dormant muses by following their intrinsic drives, listening more to their heart than their head.

## 1. Introduction

Parkinson's disease (PD) is characterized by motor control dysfunctions resulting from the degeneration of the nigrostriatal dopamine network, leading to symptoms such as tremors and paralysis [1],[2]. In addition to dopamine therapy, creative activities are frequently used to support motor function, visuospatial processing, and mental health in PD patients [3]–[5]. These individuals engage in a wide range of creative and expressive pursuits, including sewing, crafts, interior and garden design, and creative cooking. Next, everyday activities span sports and movement, fine art and design, music, and singing. Finally, patients participate in literary, scientific, technological, and performing arts, such as dance [6]. Lauring et al. [4] highlighted how dopamine therapy, along with other factors, may enhance creative motivation and artistic output in PD patients. Although PD is associated with cognitive impairments and reduced fluency and originality, patients receiving higher daily doses of dopaminergic medication tend to show greater originality [7]. Notably, those taking dopamine agonists report more frequent increases, and fewer decreases, in creativity compared with those taking levodopa alone or those not receiving dopaminergic treatments [6]. However, similar artistic enhancements have also been observed in patients with other neurological conditions, such as frontotemporal dementia, frontotemporal lobar degeneration, Lewy body dementia, and corticobasal degeneration, even in the absence of dopamine therapy [8]. Building on Lauring et al.'s [4] work and based on a creativity-development framework, the *7 Muses of the Neuro-Creative Cycle* [9], I examined how PD artists may realize their creative potential through PD-related functional and structural brain changes and psychological states/personality traits changes, in addition to through dopamine therapy. The paper first introduced the origins, components, and structure of the 7 Muses framework, which guided the review. I then examined the steps of the cycle, illustrating how neurological and psychological changes affect the 7 Muses and stimulate the emergence of creative inspiration and activity in PD artists. I concluded with a discussion of its limitations and future directions, as well as a summary and conclusion.

## 2. The 7 Muses of the Neuro-Creative Cycle

The 7 Muses of the Neuro-Creative Cycle integrated research-based theories from the creativity research pioneer Graham Wallas [10]–[14] and the father of creativity, E. Paul Torrance [15]–[19], with Kim's [20] theory of the creative climate, attitude, and thinking skills [9],[21]–[23]. The 7 Muses refer to creative individuals' attitudes, characteristics, mindsets, and/or personality traits that inspire and drive the steps of Wallas's (1926) creative cycle. Neuroscientific findings on the roles of each brain area and network, as well as on the activity and interconnectivity of creativity-related brain areas, describe the framework's inner workings and support the integrated framework [9],[21]–[23]. It was intended to develop creativity in healthy individuals. However, PD-related changes in psychological states or personality traits, as well as functional and structural brain changes consistent with the brain activity and interconnectivity involved in the 7 Muses framework, may unleash the creative potential of PD artists for greater self-expression. Some literature suggests that PD alters psychological states or personality traits, loosening inhibitions that restrain latent muses [4],[5],[24]–[27], such as openness and passion, thereby enabling PD artists to express their unique feelings and imagination beyond theoretical reasoning or technical skills [26]. Thus, disinhibited PD artists immerse themselves in creativity and build a passion for expressing their unique selves [5].

Wallas [11],[13] defined creativity as making something true to oneself (i.e., originality) while seeking to benefit others with utility/value beyond one's personal gratification. The 7 Muses framework redefined Wallas's preparation, imagination, and verification stages. The cycle was deconstructed into the following seven steps, where each of the steps is inspired and driven by each of the 7 Muses. First, in the preparation stage: During the “thinking for oneself” step, the independent muse may inspire PD patients to think for themselves and improve self-advocacy while building a sense of self and self-knowledge following a Parkinson's diagnosis. Then, during the interest-discovery step, the curiosity muse may inspire them to explore a wide variety of activities or subjects to discover what they genuinely enjoy and build interest-driven expertise, leading them to artistic self-expression as PD artists.

Second, in the imagination stage: During more spontaneous imagination, the playfulness muse may inspire a spontaneous expression of PD artists' unfiltered bottom-up feelings, sensations, and emotions. Combined with their nascent artistic skills, a playful, light-hearted, non-evaluative attitude towards mistakes and quality encourages greater output quantity, much like a hobbyist having fun while practicing a new interest. Then, during less spontaneous imagination, the confidence muse may inspire them to evaluate their output positively and seek challenges that build on their skill automaticity, much like a hobbyist consciously developing skills. Finally, during unconscious imagination, the openness muse may inspire them to relax their mind and body, open themselves to unknown possibilities, hallucinate, or loosen prior knowledge or goal fixation, and reach illumination. Illumination includes not only a-Ha! or eureka moments and original idea generation but also the therapeutic effects of trance states: Flow, hypnosis, meditation, fantasies, psychedelic assistance, and hallucinations [13].

Last, in the verification stage: During the analytic reasoning step, the interdependence muse may inspire PD artists to evaluate and improve their illuminated output and to work collaboratively for group synergetic creativity, thereby increasing the output's quality and value for targeted beneficiaries to fulfill their creative purpose. Finally, during the intuitive synthesis step, the passion muse may inspire them to commit consistent time and energy to the enjoyment of creativity and synthesize the diverse perspectives and strengths of others, thereby maximizing their creative purpose. The result is a purpose-satisfying, original and valuable creation (see Table 1).

**Table 1.** The 7 Muses, Steps, and Outcomes of the Neuro-Creative Cycle [9].

Stage	Muse	Step	Outcome
Preparation	Independence	Thinking for oneself	Self-advocacy
	Curiosity	Interest discovery	Interest-driven expertise
Imagination	Playfulness	More spontaneous imagination	Output quantity
	Confidence	Less spontaneous imagination	Challenge-seeking
	Openness	Unconscious imagination	Novel-output inspiration
Verification	Interdependence	Analytic reasoning	Output value improvement
	Passion	Intuitive synthesis	Original & valuable creation

## 3. The preparation stage: The independence and curiosity muses

Preparation is the initial stage of the creative cycle. It provides resources for the subsequent imagination stage [13]. PD-decreased frontotemporal social judgment and self-censorship may unleash the independence muse of PD artists, enabling them to focus on their own needs and desires rather than those of others, thereby improving self-advocacy. After a PD diagnosis, patients may explore a wide variety of activities or subjects to discover their interests. Then, they may accumulate interest-driven expertise (i.e., knowledge, skills, and experiences) using the frontoparietal network. Although their top-down executive function is reduced, self-chosen and curiosity-driven activities or learning can increase dopamine release, which in turn improves their memory and learning. The frontoparietal network initiates and regulates cognitive control, executing tasks that require mental effort and attention. It includes the dorsolateral and dorsomedial prefrontal cortices, as well as the posterior parietal cortex, supporting knowledge and skill acquisition [28],[29], which is essential for the preparation stage.

### 3.1. The independence muse for the thinking for oneself step: Self-advocacy

The brain tends to reinforce conformist behavior rather than encouraging independent thinking or self-advocacy. Self-consciousness occurs when social rules or norms are violated [30]. It provides immediate feedback, suppressing spontaneous behaviors due to fear of social punishment, such as embarrassment or shame [31],[32]. The dorsomedial frontal cortex processes mentalizing (i.e., theory of mind: inferring and reasoning others' thoughts, intentions, and beliefs), moral judgment, embarrassment [31],[33]–[35], and guilty feelings [30],[36]. Conformist values, beliefs, or decisions activate the ventral striatum but deactivate it when the one's values, beliefs, or decisions are inconsistent with others, like for a punishment, causing negative emotions [37],[38]. Stronger dorsomedial prefrontal cortex activation causes greater behavioral changes and less independence of mind [39]–[41]. However, dopamine therapy can disinhibit PD patients' sense of self [42], often leading to hyper-honest behavior [43],[44]. High striatal dopamine levels promote self-agency and an awareness of needs and desires aligned with the self [45],[46].

Furthermore, dopamine levels can influence personality and behavioral patterns, which social challenges and relationships can further amplify [47]. Conversely, a PD patient's personality also can affect dopamine levels [48]. A PD diagnosis initially causes panic and a sense of loss of control and insecurity, with a fatalistic realization of lost potential for pleasure in life. Patients diagnosed with the condition often recognize that they will require assistance from others to perform daily activities, which can significantly impact both their personal and professional lives [5],[49]. Their dreams and hopes shatter, and they feel anger and despair, dwell on their misfortune, ponder what to do with their enfeebled lives, and sometimes contemplate suicide [50]. Their psychological states and personality traits alter with (a) PD symptom progression as motor control fluctuates, (b) dopamine therapy-induced novelty-seeking, and (c) growing acceptance and coping skills [48],[51]. Through these changes, aspiring PD artists re-explore their unique selves and the world, eventually resolving to live for themselves rather than in despair. They can then better relate to and express their challenges, especially their feelings about their PD diagnosis and even previously uncomfortable emotions, to family and friends, even to total strangers; recognize, prioritize, and pursue what they truly want to do; and choose to live for themselves [50],[52],[53]. Moreover, PD patients show weaker dorsomedial prefrontal cortex activation [54] and mentalizing skills than healthy peers [55],[56], explaining PD artists' self-focus, than other focus, as follows: First, their reduced cognitive function, abstract thinking, processing speed, complex attention, and language skill make it difficult for them to infer and reason [56]. Second, they emotionally understand what others feel but have difficulty logically mentalizing [55]. Third, they first concentrate on their own needs and desires, then project themselves beyond themselves to mentalize others' emotions and situations [54],[57]. Lastly, freed from their outer concerns and involvement with society, they improve self-advocacy [4],[5].

Hypo-PD-frontotemporal social brain activation reduces top-down social inhibition. PD patients show ventrolateral prefrontal (e.g., inferior frontal gyrus and orbitofrontal cortex) and anterior temporal lobe atrophy, which reduces the top-down social brain's control over their behavior. Their initial anterior temporal atrophy [58] spreads across the temporal lobe areas [59] and beyond [60], reducing frontotemporal gray matter volume faster than their healthy peers [61]. Their frontotemporal atrophy or dysconnectivity and/or reduced inferior frontal gyrus volume [62],[63] reduces the self-conscious control of social concepts and norms [35]. As with PD patients' atrophied loss of function, patients with frontotemporal abnormalities, lesions, or entropy show socioemotional deficits [64]–[66]. Orbitofrontal-lesioned patients cannot appropriately respond to emotional cues (e.g., face and voice expression) and lack self-censorship [67],[68]. Frontotemporal connectivity strongly suppresses healthy individuals' social behavior through self-consciousness [67],[69]. The anterior temporal lobe and inferior frontal gyrus process all social, moral, cognitive, and emotional behaviors, including mentalizing, social judgment/evaluation, empathy, moral judgment/emotion, and social knowledge/norms [34],[70]. Orbitofrontal-lesioned patients show enhanced creativity due to their reduced top-down control or inhibition. PD patients' hypo-frontotemporal social judgment and self-censorship disinhibition enable PD artists to recognize and express their bottom-up inner states, needs, and desires, allowing them to explore ideas, activities, and means of creative expression [71]. Like those with pathologies, healthy individuals with reduced frontotemporal gray-matter volume/density over three years show enhanced creative potential [72] (which the authors did not interpret correctly). Individuals with high creative achievement typically have lower orbitofrontal gray-matter cortical volume than those with less achievement [73]. These studies suggest that healthy but less creative individuals tend towards hyper-top-down thinking and hypo-bottom-up feelings [9],[23]–[25], making them more susceptible to self-consciousness that suppresses creativity.

### 3.2. The curiosity muse for the interest-discovery step: Interest-driven knowledge/skill

Anxiety is common among PD patients [74],[75]. They tend towards pessimism, neediness, insularity, and/or apathy, driving them to higher obsessiveness, anxiety, and depression compared with healthy peers [75]–[77]. Even before their debilitating motor symptoms appear, most PD patients are (a) less extroverted, less flexible, less accepting of different opinions, less open to novelty and exploration, and less capable of coping with stress than healthy peers; (b) more routine-oriented, more fearful, more cautious, and more emotionally-unstable or paranoid [76]–[81]. According to a 40-year longitudinal study, neuroticism may precede PD diagnosis [82],[83]. Individuals with greater premorbid anxiety, depression, or impulse control disorders may develop worse PD symptoms [75],[76],[84]. High anxiety reduces their motivation to participate in activities [74], which hinders the realization of their creative potential [85]. Only when individuals feel neither too safe nor too anxious can they crave and actively explore self-growth [86],[87]. The stimuli that excite midbrain curiosity to overcome the amygdala's avoidant anxiety lead the lateral prefrontal cortex to engage in risk-taking self-growth exploration [88],[89]. Dopamine therapy reduces anxiety, regardless of motor function improvement [90]. It can reawaken dormant creative pursuits in PD patients, even if they have not been pursued for many years [4],[91],[92]. However, even those with no prior artistic experience can exhibit sudden curiosity towards creative pursuits [5],[26],[27],[93]–[98], which is more likely a result of their independence muse awakening than the effects of dopamine therapy, due to PD-related neurological and psychological changes, as described earlier. After deciding to accept their disease and live for themselves, PD patients often show a greater appreciation for life, adopting a healthier lifestyle that includes more exercise, a better diet, and a new focus in life. This leads to spending more time enjoying and establishing new, meaningful interests, activities, or projects [52]. Their disinhibited minds are open to a broader variety of topics or hobbies, with enhanced curiosity motivating them to accumulate interest-driven related knowledge, skills, and experiences [5]. The nucleus accumbens is strongly activated when engaging in self-initiated or self-chosen activities [99],[100]. Curiosity- or motivation-induced hippocampal learning and memory increase creative interest/passion [101]–[103] from an increase in dopamine [104]. More curious and open individuals exhibit higher dopamine levels [105]–[107] and stronger structural connectivity in the hippocampal areas associated with knowledge gain [108], which helps aspiring PD artists build knowledge and skills. Despite the PD-hypo-frontoparietal network's function, their curiosity- and interest-driven exploration and learning may increase their excitement and dopamine release, thereby enhancing their memory and facilitating the acquisition of knowledge and skills [109],[110].

## 4. The imagination stage: The playfulness, confidence, and openness muses

The imagination stage is an unconscious train of association that reconstructs thoughts or images from past experiences to generate original ideas or outputs [13],[111]. The preparation stage enables an imaginative mind to wander effortlessly through its vast store of memories, facilitating rapid information recall [13],[112],[113]. The cerebellum supports both conscious and unconscious physical and mental/emotional functions [114],[115]. The primary sources of the imagination stage, particularly for the unconscious imagination step, are lifetime knowledge and experiences stored in the cerebellum but not accessible to the conscious mind [116],[117]. Originality emerges from the individual's unique thoughts or experiences, or combinations of memories and images stored in the unconscious mind. It begins in the imagination, away from the group, eschewing conformity before sharing it with others to ensure its usefulness and value to them; therefore, originality must be prioritized over utility. Without an original idea, there will be nothing to evaluate or implement [11],[13],[16],[19],[118],[119].

After the preparation stage, PD artists use the more or less spontaneous imagination steps, followed by complete physical and mental relaxation, to initiate unconscious imagination, which is especially important for achieving maximum originality [13]. PD patients' hyper-default mode network activation may increase more spontaneous imagination, inspiring PD artists' playful and fun experiments with unfiltered, bottom-up feelings, sensations, and emotions combined with accumulated knowledge and experience. Familiar imagination/memory recall comes rapidly and effortlessly to mind for the idea/output generation and consumes fewer mental resources than less spontaneous imagination [111],[112],[120]. Their hypo-frontoparietal network activation may enable them to laugh at their mistakes without being focused on the goal or outcome, thereby increasing output quantity. Then, their self-positivity is amplified by PD-hyperactivation of the nucleus accumbens [5],[26],[93],[121] and hypoactivation of the dorsomedial prefrontal cortex [54]. Positive self-feedback on their output is increased by hypo-PD-frontal error-related negativity amplitude [122]. Their skill-automaticity and confidence, developed while producing high output quantities, enable them to seek greater challenges through the less spontaneous imagination step, utilizing default mode–frontotemporal network connectivity. Thus, after passionate task immersion PD artists relaxing their minds and bodies, they enter a trance statefocusing on bottom-up feelings. They thereby open themselves to unknown possibilities during incubation, loosening their frontal ties to the goals and prior knowledge, including self-related thought, while driving their cerebellar unconscious imagination to pursue the goals. The step eventually triggers illumination, which integrates prior knowledge into a novel association for an original idea (see Table 1 for a summary).

### 4.1. The playfulness muse for the more-spontaneous-imagination step: Output quantity

Original ideas and outputs spring up automatically by turning off the analytic brain, approaching problems playfully, and enabling individuals' more spontaneous imaginations to access the almost unlimited memory of their unconscious minds. The default mode network facilitates the spontaneous generation of intuitive ideas from memory, enabling the sudden emergence of numerous ideas, followed by less spontaneous imagination [120],[123],[124]. PD patients show stronger activation of the default mode network than their healthy peers, regardless of age, illness severity, or duration [63]. They maintain spontaneous thought and imagination longer [125], especially hallucinating PD patients with default mode network hyperactivation [126],[127]. Those with mild cognitive impairment show stronger within-default network connectivity than healthy peers [128]. PD artists' default mode network activation focuses their five senses on pleasurable experiences without thinking about their artistic goals. Their positive emotions promote fun and free experimentation, using their prolific ideas and desires as playthings and adding new details during each session of spontaneous self-expression without conscious effort [5],[121]. Dopamine reduces their ability to habituate to sensations [129]–[131], further promoting freer associations of ideas and artistic outputs [5],[132]. Optimal dopamine levels increase playfulness and fun [133], aided by striatal dopamine [134]. It motivates PD artists to engage in more frequent fun practice, producing more ideas and outputs while further stimulating their latent playfulness [5],[93],[95],[135]. Dopamine therapy reduces PD patients' top-down inhibitions. It increases bottom-up excitation, resulting in higher impulsivity/spontaneity than healthy peers [136], even for those with standard impulse control [137], which declines as the inferior frontal gyrus thins [138], leading some PD patients to develop impulse control disorders, like hypersexuality [139]. Growing impulsivity causes PD patients to seek instant gratification, often without regard to criticism or consequence [137]. Impulsive PD artists complete their creations whimsically, instinctively, and almost compulsively [26],[121], a result of their disinhibition of self-expression, which promotes uncontrollable self-expressive urges [5],[25],[27],[93],[140]. They emphasize self-expressive experiments and joyful art-making moments [49],[91],[93],[132],[141]. PD artists perceive what they feel as essential, such as light and color, thereby fulfilling the urge to express effusive, bottom-up feelings [26],[95],[132]. Their focus on spontaneous, unfiltered self-expression, rather than technical control and replication, reduces detailed realism and increases unfiltered simplicity, naturalness, or innocence [4],[27],[53],[132].

The analytic brain prioritizes accuracy and quality over imaginative self-expression, recycling old concepts and leaving the inhibited artist dissatisfied with their lack of originality and emotional expressiveness [49]. Reducing lateral prefrontal and orbitofrontal analytic evaluation and self-judgment enables PD artists to experiment spontaneously with novel styles of artmaking [4]. Hypo-left inferior frontal gyrus activation lowers inhibitions, enabling more flexible thinking and producing more original ideas [142]–[144]. Prefrontal atrophy, dysfunction, or deactivation can prevent self-judgment, encouraging individuals to share unusual ideas with others without fear of criticism or ridicule. Lateral prefrontal deactivation enables both creative scientists and artists to engage in more spontaneous imagination, leading to the generation of more original ideas or solutions [145]. Individuals showing diminished gray matter volume or density in the frontoparietal network experienced enhanced creative potential three years later [72]. Reduced lateral prefrontal or orbitofrontal activity and connectivity, or frontoparietal network activation, loosens inhibitory thought and behavior, like analytic judgment and self-censorship, and permits unfiltered ideas from the wild unconscious emotional brain to come into conscious awareness, stimulating original ideas [85],[120],[124],[146]. The burst in creativity requires separate generative and evaluative steps because simultaneously generating and evaluating output is like driving with feet on both the brake and gas pedals. One must develop a blind variation of spontaneous/unconscious imagination to realize originality before analytically evaluating and/or revising the product [13],[147]–[150]. A focus on high-quality production from the outset, such as a pass-fail test, often leads to disappointment with results, eroding the playful motivation for frequent practice. Good outcomes require keeping one's foot off the brake (i.e., frontal inhibition) until reaching the destination. Satisfaction with good-enough quality and the suppression of analytic judgment encourage PD artists' spontaneous bursts of output, promoting more frequent practice and a virtuous circle of skill improvement and increased output quantity [93],[95]. A focus on spontaneous, self-expressive experimentation helps [5]. PD artists can invest a considerable amount of time in their work, producing impressive output over a short period [26],[27],[95], sometimes more than one painting per week [132]. Those who occasionally sketched before their PD diagnosis can churn out multiple pastels per week thereafter, even per day [27]. Those without prior artistic experiences also often exhibit remarkable productivity [24],[25],[93],[96], from sculpture and painting to writing, as outlets for expressing their emotions about their illness and close relationships [24],[93]. Accepting good-enough quality promotes higher output quantity, which begets quality as their skills develop [151]–[153].

### 4.2. The confidence muse for the less-spontaneous-imagination step: Challenge-seeking

Playful, more spontaneous imagination inspires PD artists to continually practice their artmaking. Then, their remarkable output quantity and confidence enable them to seek challenges and refine their artistic skills [4],[93] through less spontaneous imagination. PD artists' evolving artistic styles, built on prior skill and automaticity, enable them to enhance their diverse creative skills [4],[48],[154],[155]. More-spontaneous-imagination's familiar, contextual, unfiltered, vivid, detailed sensory, imagery, or event information recall is followed by less-spontaneous-imagination's abstract information recall with evaluation for social relevance, building toward an artistic product that others could value [111],[156],[157]. Skill automaticity enables flexible shifts between unconscious and deliberate approaches to tackling and mastering challenging tasks for advanced skill acquisition [158],[159]. The failure of the default mode network to facilitate sufficient memory recall or association for challenging tasks increases the connectivity between default mode and frontoparietal networks, thereby enhancing goal-driven focus, reducing bottom-up processing and inward thought, and stimulating working memory and long-term memory [160]–[162]. Cooperation between default mode network memory-based self-generated thoughts and frontoparietal network analytic strategies and attention (a) suppresses automatic task-irrelevant thoughts or intuitive ideas; (b) intentionally recalls seemingly unrelated ideas; and (c) transforms concepts into creative ideas [112],[120],[163]. With the basal ganglia, the cerebellum develops skill-automaticity and intellectual habits, increasing cerebral functional efficiency and providing room for new knowledge or skill acquisition [164]–[166]. PD artists demonstrate better technique and a balance between tonality and luminosity, utilizing vivacious and harmonious colors. Their strokes are confident, bold, and decisive [26]. Seeing their skills improve further reduces self-consciousness, enhancing mental flexibility and higher goal-seeking behavior [5].

Skill improvement or better output does not automatically lead to confidence because the brain amplifies negative perceptions more than positive ones, magnifying those emotions with greater long-term effects. Building trust in managing difficult situations reduces negativity-amplifying perceptions [167]. Although more negative PD patients mainly complain about their pain and fatigue, the more positive focus on their preserved capabilities, adaptation ability, new coping strategies, and increased inner strength and self-respect [52]. Furthermore, PD artists' dopamine therapy activates the nucleus accumbens, encouraging positive self-feedback, boosting confidence, enjoyment, audacity, and nonconformity in their creative expressions [5],[26],[53],[93],[121]. More importantly, hypo-PD-lateral prefrontal and anterior cingulate cortices' activations reduce PD patients' error-related negativity-amplifying perception, whereas making mistakes increases healthy peers' negativity and anxiety [122], increasing PD artists' positive self-feedback on their work. Many PD artists are aware of their advancing artistic skills compared with their pre-PD diagnosis. They draw more freely and beautifully, sometimes creatively using their tremor symptoms for coloring larger areas, enhancing their creative range [52]. Their enhanced visual perception and artistic skills [26] lead them to bolder and more successful work [5],[53]. Increased pride and self-confidence enable them to present their work fearlessly to others and anticipate a positive reception [5],[26],[27],[121]. In addition to their frequent practice and suppressed self-censorship, which promotes output quantity, they share many artworks with others, some of which are valued [27]. Providing themselves with more positive feedback reinforces their confidence in creative accomplishments, motivating more frequent artistic practice and increased output quantity, innovative thinking, and, ultimately, higher output quality [5],[93],[121],[168] in the verification stage.

Confident individuals have positive self-perceptions and belief in their abilities to achieve desired outcomes, seeing positive events happening sooner and closer to them than negative ones [169]. Hypo-PD-fronto-striatal network functions increase self-positivity, unleashing the confidence muse [170],[171]. Further, hyper-PD-nucleus accumbens activation [5],[26],[53],[93],[121] combined with hypo-PD-dorsomedial prefrontal cortex activation [54] boost self-positivity, motivating productivity improvements [27] with the following neural activations: First, the ventromedial prefrontal cortex maximizes self-positivity and positive emotions [172] while minimizing negativity, reducing self-concept depreciation through its strong connectivity to other areas [173]–[175], overcoming negative self-evaluations and promoting the importance of the self [175],[176]. It guides the posterior cingulate cortex to store more self-positive and fewer self-negative memories [173],[177]. When receiving self-positive information, (ventral) medial prefrontal–posterior cingulate connectivity connects to other areas to spread the information [178]. Still, when receiving self-negative information, the connectivity deliberately recalls posterior cingulate self-positive memory [177],[179],[180]. Lastly, self-positivity reduces dorsomedial prefrontal activation that is involved in assessing self-other relationships, making moral judgments, determining guilt for social violations, and evoking social-judgment-based negative self-evaluations [33]. Weak dorsomedial prefrontal–posterior cingulate cortical connectivity causes negative anticipation of life events [178], and weak dorsomedial prefrontal–amygdalar connectivity focuses on self-negativity [181]. Social rejection causes unconfident individuals to give themselves negative feedback and to increase top-down lateral prefrontal inhibition while reducing bottom-up sensory excitation [182],[183]. They show heightened self-consciousness and mental inflexibility, including visual fixation or mental blocks [184].

### 4.3. The openness muse for the unconscious-imagination step: Novel output inspiration

Openness to unknown possibilities involves accepting uncertainty and seeking novelty. Open individuals embrace bottom-up inputs and overcome default mode network habitual thoughts and self-judgments [185],[186], thereby relaxing both the mind and body, and enabling cerebellar unconscious imagination during incubation. Detaching imagination from top-down predictions, prior beliefs, or goals following passionate task immersion plants a new goal in mind that directs the unconscious mind to search for a solution that breaks the impasse during incubation [13],[187],[188]. Hyper-PD-cerebellar activation compensates for the low striatal dopamine levels in dysfunctional basal ganglia that deplete PD patients' movement initiation and vigor [2],[189]. Regardless of their treatments and illness severity, they exhibit significantly stronger cerebellar activation in both motor and cognitive areas compared to their healthy peers [189]–[191]. The cerebellum is essential for motor imagery [192], with visual representational changes needed for illumination [193],[194]. It integrates visual perception and creative action with fine motor control and sequential memory, promoting drawing or visual arts quality [195]–[197], which supports PD patients' artmaking [4]. PD patients experience changes in psychological states and personality traits throughout the progression of the disease, becoming more open [48]. PD artists do not let their disabilities prevent them from having meaningful self-expression and often become more expressive as a result [50],[53]. Although most PD patients are the opposite of the novelty-seeking extrovert before their diagnosis, dopamine therapy brings out PD artists' latent novelty-seeking behavior. Their increased confidence and mental flexibility synergistically enhance each other, fostering freer and more open imaginations [5],[27],[53],[95]. Later-stage PD patients exhibit higher novelty-seeking and persistent self-directed behaviors [48] compared with earlier-stage PD patients [198],[199]. After immersing themselves passionately in the less spontaneously imaginative aspects of challenging tasks, their openness reduces prior beliefs or goal-driven focus, enabling whole-being relaxation. This enables passion to unconsciously guide their brains into extraordinary states, such as trances and hallucinations, and toward illumination [5],[26],[53],[91],[95],[200].

#### 4.3.1. Illumination triggered by following the heart more than the head

Whole mind-body relaxation focuses all mental faculties on present sensory enjoyment rather than on self-judgment or outcome anxiety during incubation, freeing the mind from its shackles and triggering bottom-up feelings, memories, and imaginings, including a-Ha! [201]–[203]. Illumination, like a-Ha!, appears in the mind as a surprise at the edge of chaos; the greater the surprise, the more prior information is updated [209]–[211]. The edge is where the brain transmits information most flexibly and enhances information richness [204]. It is here that bottom-up excitatory freedom exceeds top-down inhibitory control within moderate arousal levels [150],[205]. When bottom-up prediction error exceeds top-down prediction's unconscious/automatic correction threshold, the cerebellum issues an “emergency” alert of surprise, with the medial prefrontal and anterior cingulate cortices tasked to monitor and correct the error [206]. Surprise-driven knowledge updates require prior information recall to integrate with the new information, involving widespread networks and long-range frontal–temporal connectivity [207]. Lateral prefrontal–hippocampal connectivity promotes memory reorganization and consolidation only for high a-Ha!, not low a-Ha! [208]. Below the edge (i.e., hypo-surprise), the brain retains existing networks without connecting to new areas, transmitting feelings of comfortable familiarity or boredom. Still, above the edge (i.e., hyper-surprise), it issues chaotic hyperconnectivity to new areas, with no consistency between prior and new information, transmitting exciting novelty or anxiety sensations [212],[213], which can be thrilling to independent risk-takers but terrifying to dependent conformists. At the edge, the brain selects reproducible prior–new input patterns and integrates prior information into new information while maintaining general consistency [212],[213]. Using optimal-neuro-connectivity to incorporate prior information into new neural nodes maximizes the creator's and audience's pleasure and illumination [11],[214].

PD artists look at things with new eyes, beyond past stereotypical depictions, to produce more novel or pleasing images, choosing less realistic and unconventional topics, designs, and concepts [4]. They integrate their prior knowledge and skills into new materials, methods, mediums, visual themes, luminosity, and/or coloring [26],[95]. PD patients' nigrostriatal degeneration and reduced dopamine-signaling provoke their widespread lower-beta-rhythm hyper-synchrony, causing motor impairment and tremors [1], which may contribute to such integration, called synesthesia (i.e., the integration of more than one sensory system, whereby one sensory input, for example, sounds, are experienced as another, such as visual hallucinations). Some PD artists freely and intuitively integrate movement with another sense or colors, performing sequences of movements when experiencing kinetics–color synesthesia [27]. Synesthesia can also result from visual kinesthesia and visuospatial memory, compensating for PD-proprioceptive kinesthesia deficits and overreacting to visual information, even when executing motor acts [215].

Additionally, hallucinations, especially visual hallucinations, are PD-common symptoms, occurring in 46% [216] to 75% of patients [217]. They are internal imaginations that the consciousness mistakes for external reality, often accompanying strong activation of the sensory areas and/or weak cognitive control over imagination [218]. PD patients experience hallucinations due to task attention deficits resulting from (a) poor attentional shift and focus on the dorsal attention and frontoparietal networks, caused by dysconnectivity between these networks and sensory areas, which is exacerbated by ambiguous internal imagery, and (b) hyper-default mode network activation, leading to the disconnection of external visuospatial imagery from internal imagination [126],[127]. However, PD artists' passionate sensory immersion suggests hyper-focus than attention deficits. Their hallucinations can come from dopamine therapy, heightening the vividness of mental imagery and night dreaming, stimulating imagination [219],[220]. Increased dopamine levels can change perceptions, animating immobile objects, inspiring creative drives, and motivating creative artistic output [5],[95],[221].

Most importantly, PD artists' hallucinations can come from whole-being relaxation through their openness to unknown possibilities that reduce their top-down frontal control and increase their bottom-up unfiltered, intense, vivid feelings, emotions, and imaginings, triggering surprise. Some experience Zen-like [5],[26],[91] feelings of being in the best place [53]. Their peaceful, holistic mind-body relaxation frees them from PD's physical and mental burdens to feel fulfilled and to experience the most illuminating moments of their lives, despite their degenerative condition [26],[53],[95],[200]. Creativity is about expressing and authentically connecting with the self, reflecting the creator's authentic thoughts, emotions, and preoccupations, especially when facing a life-changing illness [49]. The deeply immersed selves of PD artists have a strong need to express their feelings and emotions freely in their work [53]. They feel a sense of control in their creative process despite their frustrations with PD symptoms in all other aspects of their lives [95]. In their emotional refuge, motor control improves, reducing tremor symptoms and enabling stronger, more fluid artistic movements [26],[95],[132],[200]. It sublimates their despair of living with antisocial PD-related symptoms to the joy of creative expression [3],[5],[25],[141].

## 5. The verification stage: The interdependence and passion muses

Illumination is the starting point of an extended, often painful verification to turn illuminated novel/original output into valid/valued products [11],[13]. Verification integrates analysis and intuition to evaluate the quality and value of the illuminated output, synthesizing diverse strengths and perspectives [13],[123],[222],[223]. Rigorous, analytical, and logical self-evaluation and evaluation from others are necessary for the verification of illuminated output [13],[151],[224]. The greater the cooperation between individuals' default mode and frontoparietal networks, the more successful their verification [123]. During the analytic-reasoning step, the interdependence muse increases frontotemporal social brain and frontoparietal network activations to collaboratively evaluate the illuminated output for improvements in usefulness and value. Through interdependence with others' feedback and collaborative synergy, PD artists analyze the illuminated output for enhancements, focusing on the work's potential benefit to society. Next, during the intuitive synthesis step, the passion muse activates the addiction network, committing to bottom-up synthesis and vividly imagining the output's future impact on its target audience. This process enhances the perceived value of the production until the final creation is ready for presentation. PD artists dedicate their passion to fulfilling their purposes and commit consistent time and energy to continually improve the quality of their novel/original output. Collaborative group participants leverage each other's strengths to seek synergetic creativity through shared belonging, commitment, and social significance of the creative endeavor. The enjoyment of creativity leads to original and useful creations through the repeated activation of the addiction/passion network.

### 5.1. The interdependence muse for the analytic-reasoning step: Output value improvement

After individuals deactivate the social-brain areas to prioritize the self, individuality, and independence, they re-activate them for other orientations and collaborative synergy [11],[13]. Humans have an inherent desire for social belonging. During rest and social reflection, the social brain becomes activated, facilitating self-reflection on social interactions [225],[226]. The means for independence's authenticity combined with interdependence's empathetic communication to enable individual/collaborative artistic, scientific, and technological advancements include (a) leveraging the tension than harmony between different ideas or thinking types among collaborators, like analytic and intuitive thinkers; and (b) understanding and meeting others', especially the audience's, needs and desires when trying a new idea, approach, or product [118],[222],[227], which is why the independent muse must come before the interdependent muse [13].

After a PD diagnosis, many PD patients are encouraged to (re)engage in creative activities, which serve as emotional outlets and help them mitigate feelings of despair [5],[27],[121],[132]. As they unleash the interdependence muse through better life-focus or artistic pursuits, they report improved relationships with family, friends, and/or caregivers and rate their quality of life as good or excellent [52]. More frequent engagement in creative activities elevates dopamine and oxytocin levels [228],[229], delivering pleasurable rewards that further promote their socioemotional bonding and interdependence [230],[231]. Artistic creativity requires artists to possess emotional knowledge and sensitivity beyond their purely technical skills for their creations to impact an audience [200],[232]. Less successful artists tend to worry more about realism and detail [233]. However, PD artists tend to express their inner selves' positive thoughts and feelings through joyful artmaking, using a bolder, warmer, brighter, and more vibrant palette [26],[53],[93], which evokes positive emotions in viewers [234],[235]. Such work connects with their audience, increasing its perception of originality [27],[236]. One PD artist/author's literary skills and emotional sensitivity produced a literary prize-winning historical book, and another PD artist/poet who composed international award-winning verse was invited to broadcast writings on the radio [98]. PD artists continually enhance their skills, learning from both their audiences' feedback and interactions with fellow artists in the studio, even as their physical limitations worsen [5],[26]. Although some of their output is criticized, their hypo-front-striatal network function [170],[171], hypo-PD error-related negativity-amplifying perception [122], and already-developed muses reduce critical self-censorship. They view the feedback constructively, maintain their confidence, and incorporate it into their work to improve their creative skills and enhance the value of their output [4],[27],[121],[132]. By sharing their work publicly, PD artists enrich others' experiences, challenging public perceptions of PD patients as helpless dependents, re-establishing their sense of self-worth, and boosting their motivation, confidence, and joy [237],[238]. Developing creative strengths leads to life fulfillment, especially when sharing their strengths with others [239],[240]. Although artmaking itself is meaningful to them regardless of any potential financial gain, they sometimes achieve commercial success [27],[121],[132]. They find meaning in their work through personal and social rewards, feeling that they have a purpose, not just a job, that positively impacts the world or inspires others [5],[26],[50]. Some PD patients also find purpose in assisting with fundraising events or sharing their journey, thereby raising awareness about PD through podcasts, radio and TV interviews, and personal connections [52].

### 5.2. The passion muse for the intuitive-synthesis step: An original and valuable creation

When individuals enjoy what they do, dopamine helps them focus their minds to work hard until they achieve mastery [241],[242]. Passion provides intrinsic, not extrinsic (e.g., monetary reward or others' praise) motivation. It increases midbrain and ventral striatal activations, especially of the nucleus accumbens [105],[243],[244]. The higher the passion, the stronger reward/dopamine network activation, inspiring greater creative pursuits [229]. The creative achievements of individuals with global neuro-interdependence occur when their top-down knowledge and goals are synchronized with their bottom-up passion. It enables them to share with others the meaningfulness of their work and purpose, using the knowledge about others via vivid imagination to increase the amount of pleasure in the world [11],[13]. Both thinking and feeling, driven by bottom-up passion, enable them to overcome hyper-top-down goals and fears about the acceptance of their artistry [11],[13]. Their love is like an addiction, with neither a desperate eagerness to prove themselves to others that could wear out their mental energy and health, nor a goal-driven anxiousness that prevents global-neuro-connectivity [13]. Their consistent commitment to a passion for improvement fosters reward sensitivity, heightening reward-cue-driven enthusiasm and euphoria, and motivating them to realize their creative potential [13],[208],[245],[246]. Creative individuals show high dopamine-receptor density [5],[247]–[252], strong reward network activation [246], and heightened reward sensitivity (i.e., sensitizing the brain's cravings) [85],[158],[253], even at rest [254]. Passionate individuals' reward networks amplify favorable anticipations and suppress negative outcome-related emotions [255]–[257]. Extraordinary anticipation sustains dopamine release, exaggerating the reward value and sensitizing cravings without regard for the pleasure of the actual reward outcome [241],[258]. Their heightened reward sensitivity produces excessive cravings, maintaining both passion and addiction, soaring above and beyond the remembered, anticipated, and experienced pleasurable value of a repeated outcome [241]. Passion sustains the nucleus accumbens core's dopamine release [259], central to powering creative individuals' [228],[229] drive for long, challenging endeavors to obtain rewards. Outcome reward activations originate from the orbitofrontal cortex, supporting reward sensitivity for creative thinking [254]. The orbitofrontal cortex considers not only goal-relevant values but also personal values unrelated to immediate practical benefits [260],[261]. Reward-sensitive individuals' orbitofrontal activation even before a-Ha! expresses their enjoyment of creativity regardless of the outcome reward, and orbitofrontal hedonic hotspots–striatal connectivity updates reward values and drives future a-Ha!-seeking behavior [245]. Thus, what develops and sustains creative passion is a heightened reward sensitivity. Individuals are drawn not merely to the outcomes of their creative work but to the process itself—consistently engaging in creative pursuits because they find the act of creating inherently rewarding.

Dopamine therapy increases PD patients' dopamine receptor density [262] and heightens a reward sensitivity [154]. Dopamine boosts PD artists' creative drive as well as the rewards from their creative endeavors [4],[5],[168],[121],[262]. It makes them more reward- and less punishment-sensitive [5]. Punishment-sensitive individuals curtail their explorative behavior [88],[263]. Yet PD artists often have hyper-pre-PD euphoric–dysphoric expressiveness, and dopamine therapy heightens their already expressive emotionalism [264] but reduces their negative emotional intensity, regardless of their bottom-up input awareness or cognitive ability [265]. PD artists express intense, unfiltered emotions and are more energetic and enthusiastic than other PD patients, providing energy to create [5],[95]. However, it is their enjoyment of creativity that drives them to continue enhancing their creative skills and improving the quality of their output [94],[140]. They adopt abstract [53],[93],[95] or impressionist [132] styles for self-expression of their life meaning, contributing to intuitive synthesis. They also synthesize others' diverse perspectives and strengths with their bottom-up intuition, expanding their imagination to see the holistic picture and abstract essence beyond just focusing on the details, as opposed to realism artists who frequently copy others' compositions. They may have technical skill, but they lack the passion or originality PD artists commonly exhibit [5]. Their consistent time and energy commitment to the enjoyment of creativity, further heightening reward sensitivity, may intensify an addiction-like passion, eventually leading to original and valuable creations.

They often sacrifice their social life, interests, and responsibilities for their artistic pursuits [93]. They may temporarily go without food to pursue self-defining creative work, as practice, pleasure, and passion for art promote work of increasing quality, positively addicting them to creative pursuits [5],[24],[53],[91],[93],[95],[96],[121],[132]. They pursue their passion for days and sometimes sleepless nights, often as an escape from PD-related sleep disorders [53],[95],[121],[132], partly because their reduced frontotemporal cortical surface area causes nighttime fidgeting or frequent distressful dreams [266]. Their commitment to artmaking, despite disrupting other needs, even sleep, continues a virtuous cycle of creative output quantity, quality, and passion [26],[53],[95]. Their love for the creative process itself transforms their emotional refuge from the distress of their condition into a creative haven [5],[26],[27],[53],[91],[121],[132]. Their passionate sensory immersion in what they love can make their tremors completely abate [26],[49],[95] as they become deeply attached to their creativity, a source of self-discovery and socially recognized personal enrichment, to the point where it becomes an essential part of their self-identity as creative artists [4],[5]. Channeling their energies into creative endeavors not only brings meaning to themselves but also inspires hope, even as they become increasingly dependent on others for basic life needs [50],[267]. Although many PD patients show depression due to the effects of the degenerative disease impacting their quality of life, changing how they feel and think about the future, those with higher passion, immersion, and optimism show better health [76],[83],[268]. PD artists' intense passion for self-defining creative work drives them to overcome challenges in committing to their creative process [132],[269]. It improves their brain functions and plasticity beyond creativity [270]–[272]. It enhances their mental health while giving them a sense of accomplishment in overcoming the walls of despair associated with their disease [26],[50].

## 6. Limitations and future directions

Creative engagement plays a vital role in PD care, serving both as a coping mechanism and a rehabilitative tool that extends beyond artistic expression [52],[275]. PD patients often experience tremor reduction during voluntary or externally guided movement and hand-based activities, such as grasping and object manipulation, which eases their motor symptoms [274]. Many PD patients report increased creativity in visual arts, design, movement-based activities, and writing [52]. Notably, those with no prior artistic background discover new talents after diagnosis. Families and caregivers should encourage PD patients to explore familiar as well as unfamiliar activities, which may lead to unexpected joy and self-discovery, such as PD artistry. Creativity offers a powerful way to live meaningfully, uplifting the spirit and empowering individuals to break free from limitations and explore new possibilities. It supports emotional expression, strengthens social bonds, and helps preserve a sense of identity beyond the disease, improving physical and mental health and overall quality of life [71],[275]. For these reasons, creativity should be a central component of PD care. Anxiety, which is common among PD patients, can hinder their ability to cope with the disease. Early assessment of psychological states and personality traits is critical for delivering personalized care [75]. Additionally, identifying patients' latent interests early enables the development of tailored creative therapies that unveil hidden potential.

This paper has several limitations. First, most reviewed studies employed observational or correlational methods rather than experimental designs, which limits the ability to draw causal conclusions regarding PD patients' potential for creativity development. Experimental research and longitudinal studies are necessary to validate these findings and track creative changes over time, providing insights into disease progression. Personalized monitoring of creative expression may also enhance care strategies by supporting emotional resilience, social connection, and identity preservation [71],[275].

Second, the generalizability of current findings is limited by regional and cultural representation. Most studies have been conducted in Western countries (e.g., Russia, Europe, the Americas, Australia, and New Zealand), while research from Eastern regions (e.g., Asia and the Middle East) remains scarce and often involves smaller sample sizes [273]. This imbalance may obscure the understanding of how cultural context influences creative engagement. Additionally, PD appears more prevalent (a) in Western than Eastern countries, (b) as age increases across both regions, and (c) in males, especially in Western populations [273]. Future studies should include underrepresented populations, especially in the East.

Third, social background and gender may shape how PD patients engage with creativity and express their 7 Muses. For instance, women more frequently report creative changes, yet this remains underexplored in both research and treatment [52]. In future studies, researchers should examine these social factors more closely.

Last, most researchers focus on artistic creativity. However, creativity is essential across all domains of life. Thus, researchers should broaden the scope to investigate how a wider variety of creative fields, such as scientific, technological, and everyday creativity, affects the lives of PD patients.

The 7 Muses framework offers a valuable lens for understanding how creativity emerges in PD artists and beyond. Applied more broadly, the framework can help refine the Neuro-Creative Cycle and support individuals in realizing their creative potential, advancing Wallas's vision of widespread creative contribution and commitment to social progress [52].

## 7. Conclusions

While dopamine therapy is well-documented for disinhibiting creativity in PD artists, I highlighted that a broader constellation of factors shapes how PD fosters creativity. Functional and structural brain changes, along with shifts in psychological states and personality traits, also play a critical role in nurturing creative expression. Many PD patients reflect profoundly after diagnosis, asking themselves, “How do I want to spend the time I have left?” Their introspection often leads to a more meaningful life, guided by personal values and creative engagement—a transformation relevant not only to PD patients but to anyone seeking purpose in the face of life's uncertainty. Their neurological and psychological changes may help unleash the 7 Muses of the Neuro-Creative Cycle: independence, curiosity, playfulness, confidence, openness, interdependence, and passion [9].

In the preparation stage, during the thinking-for-oneself step, PD patients' life-focus shifts after diagnosis, and their frontotemporal social brain hypoactivity may reduce social judgment and self-censorship, unleashing the independence muse, which helps them break away from conformist pressures and enhance self-knowledge and self-advocacy. Next, during the interest-discovery step, their life-focus shifts help them pursue what they genuinely enjoy after exploring a wide range of activities, unleashing the curiosity muse, which enables them to build interest-driven expertise despite their reduced frontoparietal executive functions.

In the imagination stage, during the more spontaneous imagination step, default mode network hyperactivity and frontoparietal network hypoactivity facilitate experimentation and acceptance of mistakes, unleashing the playfulness muse, which enables PD artists to increase output quantity without worrying about their output quality. Next, during the less spontaneous imagination step, increased output quantity, reduced frontal error-related negativity, and dorsomedial prefrontal hypoactivity encourage positive self-feedback, unleashing the confidence muse, which enables them to seek new challenges and build new skills based on improving existing skill automaticity. Finally, during the unconscious imagination step, prefrontal hypoactivity, cerebellar hyperactivity, and dopamine-driven novelty-seeking loosen prefrontal ties to previous knowledge and goals, unleashing the openness muse, which enables incubation and unveils unknown possibilities, triggering illumination.

In the verification stage, during the analytic reasoning step, feedback and collaboration help reactivate the frontotemporal social brain, unleashing the interdependence muse, which enables PD artists to improve the quality and value of the illuminated output for future audiences. Finally, during the intuitive synthesis step, the integration of diverse perspectives and sustained emotional investment and engagement builds their self-identity as creative artists, unleashing the passion muse, which enables them to persist until they finish an original and valuable creation.

PD artists navigate the creative cycle, consisting of preparation, imagination, and verification stages, through a dynamic balance between top-down goal-driven and bottom-up emotion-driven processing. In the preparation stage, they rely on top-down control to learn and remember, thereby building expertise. However, in the imagination stage, they shift toward free bottom-up sensory flows, which foster spontaneity and originality, especially at the edge of chaos, where originality is prioritized over utility [13],[19],[118]. This shift helps PD artists overcome the inner critic and self-censorship that often limit the self-expression of non-PD artists, giving them an edge in authenticity and emotional depth. Illumination, the creative breakthrough, usually arises not from logic alone but from following the heart. In the verification stage, they return to top-down analysis to refine their work yet finalize it using bottom-up intuition. As PD artists strengthen their artistic abilities, they fuel their passions, driving new creative cycles of continued pursuits, enabling them to realize their creative potential, often with astounding results.

## Use of AI tools declaration

The author declares she has not used Artificial Intelligence (AI) tools in the creation of this article.
